# Editorial: A wonder legume, soybean: prospects for improvement

**DOI:** 10.3389/fpls.2023.1294185

**Published:** 2023-10-26

**Authors:** Ayyagari Ramlal, Aparna Nautiyal, S.K. Lal, Godfree Chigeza

**Affiliations:** ^1^ School of Biological Sciences, Universiti Sains Malaysia (USM), Georgetown, Malaysia; ^2^ Division of Genetics, Indian Council of Agricultural Research (ICAR)-Indian Agricultural Research Institute (IARI), New Delhi, India; ^3^ Department of Botany, Deshbandhu College, University of Delhi, Delhi, India; ^4^ Soybean Breeder, International Institute of Tropical Agriculture (IITA) Zambia, Lusaka, Zambia

**Keywords:** soybean, breeding, crop improvement, wonder legume, agricultural and industrial crop

*Glycine max* (L.) Merr. (soybean) is an economically, agriculturally and industrially (food, paper, textile, pharmaceuticals) important crop on a global scale. It is often referred to as a ‘wonder legume’ and ‘gold from the soil’ due to its substantial value and versatile nature. Soybean seeds are rich in proteins and serve as a vital source of macronutrients, micronutrients, vitamins, and minerals. It holds a crucial role as a multi-purpose crop, serving both as animal feed and as a staple in human diets (Song et al., 2023). Soybean is also a medicinal crop and harbours diverse compounds known for their therapeutic potential (Ramlal et al., 2022a; Ramlal et al., 2023a). Traditionally, soybean has been cultivated using conventional breeding methods, driven by the necessity to enhance production to meet the growing population’s demands which is a time-consuming and tedious process (Maranna et al., 2016). Breeding endeavours also focus on elevating the quality of proteins and oils while concurrently reducing anti-nutritional components.

Soybean, owing to its extensive genetic variability and possession of significant agronomically important traits, shows immense potential with different applications such as genetic mapping, marker development and genome-wide association mapping. These avenues facilitate the identification of key genes related to important traits and significantly contribute to advancements in soybean breeding (Yoosefzadeh-Najafabadi et al., 2022; Du et al., 2023; Song et al., 2023). Moreover, the rich genetic diversity of soybeans proves crucial in investigating heterosis and male sterility, fundamental for breeding initiatives (Ramlal et al., 2022b; Farinati et al., 2023). Additionally, it plays a vital role in weight prediction using image-based machine-learning techniques (Duc et al., 2023). Nonetheless, it’s important to acknowledge that soybean faces substantial challenges due to climate change, making it susceptible to both biotic and abiotic stressors that significantly impair yield and production (Jiang et al., 2023). For instance, soybean is particularly vulnerable to waterlogging during critical growth stages such as germination, emergence, and grain-filling, resulting in considerable productivity losses (Rajendran et al., 2022; Rajendran et al., 2023). Similarly, the development of haploid and doubled haploid lines holds key insights into various mechanisms and genetic mapping, advancing research in soybeans (Ramlal et al., 2023b). Therefore, comprehending these factors limiting yield is imperative for successfully crafting improved soybean varieties with desired traits.

A review of high-impact articles providing evidence and insights on agriculturally and industrially important crops is essential such as soybean that will eventually contribute to effective approaches to the development of climate-smart food crops to meet the food and feed requirements of future generations for a sustainable environment.

Niazian et al. have devised a significant biotechnological screening protocol using the *RUBY* reporter system in soybean, enabling *ex vitro* and *in vitro* hairy root transformation (HRT) through *Agrobacterium rhizogenes*. Optimization of various parameters was done and their findings indicated that the *in vitro* approach is more efficient in inducing hairy roots and transformed roots (expressing RUBY) compared to the *ex vitro* method. However, the *ex vitro* approach was identified as simple and rapid. The highest transformation of RUBY was achieved using 7-day-old cotyledons of cv. Bert. The optimized parameters also resulted in the highest RUBY expression using a two-step *ex vitro* HRT. Moreover, employing machine learning, they further assessed and validated their optimized protocols for both assays. This study is of great significance as it establishes an efficient and dependable protocol for HRT, a crucial tool for functional studies in soybeans.

Seed longevity is a significant challenge in crops like soybeans due to their genetic composition and prevailing environmental conditions. Factors such as seed coat characteristics, compromised DNA integrity, dysfunctional ribosomes, lipid peroxidation and an inadequate antioxidant system contribute to the issue of seed longevity in soybeans. Variability in seed longevity among different genotypes is influenced by various parameters including seed coat proportion, cell structure, calcium and lignin content, pore density, and the space between the seed coat and cotyledon. According to Rao et al. effective strategies for soybean storage should encompass a robust antioxidant system, ample protective proteins, proficient nucleotide and protein repair mechanisms and mechanisms for scavenging free radicals. Identifying molecular markers and quantitative trait loci (QTLs) associated with these mechanisms is vital for enhancing seed longevity in soybeans. Rao et al. have extensively reviewed seed longevity and vigour in soybeans, employing a comprehensive approach that integrates morphological, biochemical and molecular aspects. Their insights also provide valuable guidance on optimal harvest, processing and storage practices, offering significant contributions to breeding programs focused on improving soybean seed longevity.

To boost agricultural productivity, especially in crops rich in proteins, it is essential to gain a deep understanding of the genetic basis underlying protein content (PC). In the study conducted by Zhang et al., a comprehensive genome-wide association study (GWAS) was carried out to identify QTLs associated with PC in soybean. This investigation involved the analysis of 264 re-sequenced soybean accessions and utilized a high-quality single nucleotide polymorphism map. The study uncovered 11 QTLs that displayed significant associations with PC. Noteworthy qPC-14, a QTL, was identified through GWAS in both Sanya and Nanjing cities of China and exhibited clear evidence of strong selection during the process of improving soybean varieties. Moreover, the research identified 15 candidate genes located within qPC-14, and notably, three of these candidate genes displayed distinct expression patterns between high-PC and low-PC soybean varieties during the seed development stage. These identified QTLs hold immense promise as valuable resources for future molecular breeding studies and programs aimed at enhancing soybean varieties with higher protein content. The candidate genes associated with these QTLs will play pivotal roles in unravelling the intricate mechanisms that govern the biosynthesis of proteins in soybeans.

Kumar et al. have focused on the QTL investigation associated with both 100-seed weight and shape in soybeans. The size and shape of soybean seeds hold significant importance, particularly for specialized uses like tofu, natto, miso and edamame. The study utilized a mapping population derived from the crossing of grain-type and vegetable soybeans. This analysis successfully emphasized the most influential genomic regions that govern seed size and weight in soybeans. Moreover, the study proposed specific candidate genes that play a regulatory role in determining these pivotal seed attributes in soybeans. The insights from this study are anticipated to significantly contribute to marker-assisted breeding efforts aimed at developing soybean varieties with enhanced seed weight and the desired seed shape.

The study by Nawade et al., carried on to the DMP (DUF679 membrane protein) family successfully identified and characterized 14 genes in soybeans using multiple parameters. Additionally, the study involved a thorough investigation of tissue-specific and stress-responsive expression patterns of those genes. To gain further insights into the evolutionary relationships of this gene family, the authors have constructed a phylogenetic tree and concluded that GmDMPs had gone through purifying selection during their evolution. The authors concluded that their study offered a comprehensive overview of the soybean GmDMP family and paved the way for further investigations and analyses of DMP genes in soybeans and other crop species.

In a pioneering effort, Guo et al. conducted an investigation of the kip-related proteins (KRPs) gene family of soybeans. KRPs play a pivotal role in plant growth and development by modulating through inhibition of the cyclin-dependent kinases (CYC-CDK) complex, thus effectively regulating cell cycle progression. They have identified nine GmKRP genes in soybeans through the bioinformatic approaches. One of the key findings highlighted that GmKRP2a, which is situated in the nucleus, significantly contributes to root development by effectively regulating cell cycle progression. RNA-seq results further emphasized its involvement in cell cycle regulation through its influence on ribosome regulation, cell expansion, hormone response, stress response, and plant pathogen response pathways. This study stands as an intriguing exploration with immense potential for broader applications, which could be further investigated in detail across various crops, including soybeans.

A concise review of this subject emphasizes the paramount applicability of soybeans, both as a crucial agricultural and industrial crop. The research contributions highlighted several key aspects that significantly impact the soybean domain. First, the extended viability of soybean seeds is underscored as a critical factor for improved agricultural output, as emphasized by Rao et al. Niazian et al. introduced a groundbreaking RUBY reporter system for efficient visualization of hairy root transformation, a pivotal advancement in soybean research. Additionally, understanding the genetic basis of seed weight, shape, and protein content, as explored by Kumar et al. and Zhang et al. respectively, holds immense promise for refining breeding strategies. Furthermore, the roles of the DUF679 membrane proteins family genes, as elucidated by Nawade et al. and the kip-related proteins (KRPs) gene family, as comprehensively studied by Guo et al. shed light on critical molecular components vital for various aspects of soybean growth and development. Altogether, these studies collectively widen the scope for exploring the multifaceted potential of soybeans. The wealth of knowledge obtained from this research will undoubtedly fuel new, advanced studies and breeding strategies, propelling further enhancements in soybean cultivation. It is essential to recognize the synergy between theoretical research, varietal development and field-related studies of agriculturally important crops like soybeans. Gratitude is extended to the journal editors, peer reviewers and authors for their invaluable contributions to this volume, ultimately enriching the understanding of soybeans.

## Author contributions

AR: Data curation, Resources, Writing – original draft. AN: Conceptualization, Investigation, Project administration, Supervision, Validation, Writing – review & editing. SL: Conceptualization, Investigation, Project administration, Supervision, Validation, Writing – review & editing. GC: Conceptualization, Investigation, Supervision, Validation, Writing – review & editing.
